# Pernicious Anæmia

**Published:** 1890-02-15

**Authors:** 


### PERNICIOUS ANÆMIA.

In the "Retrospect" for November 16th, we referred to Dr. William Hunter's observations on the watery excretions of pernicious anæmia, in which the presence of pathological urobilin in great quantity is regarded as a diagnostic sign. We then remarked that "until further investigation is made, it may perhaps be permissible to regard the high coloured urine observed by Dr. Hunter in this one case as an accidental occurrence." Dr. William Hunter has since continued his articles in the *Practitioner*, and in that journal for December he sums up his observations as follows. Three changes are made out sufficiently well to merit attention: 1. The presence of pathological urobilin in great quantity in the urine, derived in all probability partly from the bile pigments in the intestinal tract; partly from other products of hœmoglobin destruction. 2. The presence of blood-pigment, recognisable on microscopical examination. 3. An increased excretion of iron. All these changes, Dr. Hunter says, point to the existence of one condition of blood, namely an excessive destruction. In addition to this pathological significance, Dr. Hunter thinks the changes are of no little importance from a diagnostic point of view. But unfortunately, as Dr. Hunter himself admits, "the urine in pernicious anæmia may not always show these well marked, and when present, characteristic signs." Dr. Hunter, however, adds that such signs will be found at some period or other of the history of a case of pernicious anæmia, especially when the patient is not gaining ground, or in other words, when there are "aggravations of weakness." In his papers the author has endeavoured to show that this blood destruction is initiated in the portal system, and depends upon changes occurring in the gastro-intestinal tract. It does not appear, however, that what these changes are has been clearly indicated. Anæmia is frequently connected with fatty or lardaceous deposits in the intestinal structures, but when such changes are present there is diarrhœa, most commonly white diarrhœa, and this drain on the system is sufficient to account for the anæmic condition. But so-called pernicious anæmia frequently exists without any accompanying diarrhœa. No doubt the changes in the blood and organs so ably illustrated by Dr. Hunter do occur, but the cause thereof has not yet been demonstrated. Very similar changes may occur in the blood from the effects of heat, as portrayed by Dr. Lauder Brunton. And it is quite probable that various influences may produce similar effects.

